# Epidemiology and Clinical Features of Hydatid Cyst in Northern Iran from 2005 to 2015

**Published:** 2018

**Authors:** Parisa ISLAMI PARKOOHI, Marjan JAHANI, Fatemeh HOSSEINZADEH, Shokufeh TAGHIAN, Forugh ROSTAMI, Abdollah MOUSAVI, Mohammad Sadegh REZAI

**Affiliations:** 1. Dept. of Community Medicine, School of Medicine, Mazandaran University of Medical Sciences, Sari, Iran; 2. Student Research Committee, Mazandaran University of Medical Sciences, Sari, Iran; 3. Pediatric Infectious Diseases Research Center, Mazandaran University of Medical Sciences, Sari, Iran

**Keywords:** Cystic echinococcosis, Prevalence, Clinical features, Iran

## Abstract

**Background:**

Human hydatid disease imposes significant impacts on public health by producing substantial morbidity and mortality in involved communities. This study aimed to evaluate the epidemiology and clinical features of hydatid cyst in northern Iran as a breeding center for the infection.

**Methods:**

In this cross-sectional study, the hospital records of all hydatidosis-affected patients admitted in three teaching hospitals of Mazandaran Province between Mar 2005–2015 were reviewed. Hydatidosis-relevant demographic characteristics, clinical findings, and laboratory data were collected. The descriptive statistical analysis was performed by SPSS software.

**Results:**

Totally, 79 patients with the mean age of 42.00±23.82 yr were admitted with cystic echinococcosis (CE) diagnosis. Moreover, the highest and the lowest prevalence of CE cases were in age ranges of 50–59 (19.0%) and more than 80 (5.0%) yr, respectively. Male/female ratio was 0.88 (47.0% vs. 53.0%). Majority of the cases were urban residents (54.0%) and had no close contact with animals (58.0%). Nearly, two third of the patients (n=54), the affected organ was liver. The diameter of the cysts was variable from 2 to 15 cm. Most of the patients had a single hydatid cyst. Four patients were diagnosed as secondary hydatid cyst. Medical treatment with antiparasitic agents was done for 47 individuals and in 7 cases; it was the only treatment approach. Percutaneous puncture-aspiration-injection reinjection (PAIR) technique was applied for 6 cases. Sixty-six patients underwent radical surgery. No data was available on eosinophil count or serological tests.

**Conclusion:**

CE is approximately prevalent in Iranian population. Development of new diagnostic methods and therapeutic procedures is worthy. Moreover, it is necessary to design and develop a registry and surveillance system by a multidisciplinary team.

## Introduction

Cystic echinococcosis (CE), hydatid disease and/or hydatidosis is a parasitic infestation caused by the larval cystic stage of a small taeniid-type tapeworm called *Echinococcus granulosus* that may cause illness in intermediate hosts, generally herbivorous animals and people infected accidentally. Hydatidosis is a generally chronic endemic helminthic zoonotic disease in human (1). The lifecycle of this parasite is between livestock as the usual intermediate hosts and dogs as the definitive host (2). Human is infected as an accidental intermediate host and the relevant infection is known as hydatidosis. Seventy percent of cysts are caught in the hepatic sinusoids. A few ova pass through the liver parenchyma and are held up in the lung and systemic circulation. Therefore, the main affected organs are the liver and lungs (3). The majority of hydatidosis cases are asymptomatic at the early stages of the infection and become symptomatic suddenly when the cyst is being larger or complicated (4).

Human hydatidosis imposes significant impacts on public health. It continues to produce substantial morbidity and mortality as well as economic losses in communities involved (5). This health problem has been addressed within the WHO’s 2008–2015 strategic plans for control of Neglected Tropical Diseases (NTDs) (6).

Recently, several studies have been conducted in various settings by many health sectors to complete the epidemiologic profile of the hydatidosis in order to appropriate recognition, priority setting and funding for research, prevention, and control programs (7).

Hydatidosis has a global distribution with worldwide annual incidence rate of 1–200 per 100000 (8). It is endemic in many sheep and cattle-raising geographic areas including Mediterranean countries, the Middle East, Eastern Europe and South America (9). In Iran, the hydatidosis is being actively transmitted and its annual incidence rate is estimated 0.61 per 100000 (10). The ongoing surveys have investigated the medical and monetary burden of hydatidosis in Iran (11, 12).

Since the epidemiological study of hydatidosis was not conducted in north of Iran, especially for 10 yr, we aimed to evaluate the epidemiology and clinical features of hydatidosis, in three referral educational hospitals in north of Iran.

## Materials and Methods

This cross-sectional study was performed during ten-years, from Mar 2005–2015 on patients admitted to medical and surgical wards of three teaching hospitals of Mazandaran Province including Imam Khomeini, Bouali Sina and Razi Hospitals.

Patients whose hydatidosis diagnoses were ruled out following diagnostic workup, surgery or pathologic investigations were excluded from the study. Discharge diagnose was used to identify hydatidosis cases. Hydatidosis was diagnosed based on clinical manifestation, imaging and laboratory parameters.

Data were collected from hospital records. The demographic data consisted of age, gender, location (urban or rural), and possible contact with dogs or domestics. The clinical-related data were signs and symptoms, treatment approach (medical, surgical or both), prescribed anti-parasitic agents as well as cyst details (number, localization, and size) obtained from reports of diagnostic imaging modalities including ultrasonography, computed tomography (CT) scan and magnetic resonance imaging (MRI). In addition, some laboratory data such as eosinophil count was checked. As we studied patients’ records, confidentiality of the patients’ information was preserved.

For analysis, the localization of cyst was categorized into 4 groups of hepatic, pulmonary, hepato-pulmonary and extra hepato-pulmonary cysts. Moreover, the chief complaints were considered as symptoms which classified into five categories including gastrointestinal symptoms (mainly nausea and vomiting, abdominal pain and hepatomegaly), pulmonary symptoms (mainly cough, dyspnea, and chest pain) and neurologic symptoms (seizure), urologic symptoms (flank pain) and others.

The statistical analysis was carried out using SPSS software for windows ver. 19.0 (Chicago, IL, USA). Absolute or percentages were used to describe categorical variables. For quantitative variables, depending on the distribution, results were expressed as mean±standard deviations (SD) or median values and inter-quartile ranges (IQR). A *P*-value less than 0.05 was considered being statistically significant.

## Results

From March 2005–2015, 79 patients with age range of 1 to 88 yr and average of 42.00±23.82 yr were admitted with hydatidosis diagnosis. The average ages in male and female patients were 42.20±23.42 and 41.80±23.44 yr, respectively. The most prevalent cases were urban women (Table 1).

**Table 1:** Characteristics of individuals (n=79) with a hydatidosis

***Variable***		***N (%)***
Gender	Male	37 (47)
	Female	42 (53)
Location	Urban	43 (54)
	Rural	36 (46)
History of close contacts with dogs or domestic animals	Yes	21 (27)
	No	46 (58)
	Not specified	12 (15)

Eleven cases had positive history of close contact with animals and majority of them had no close contact.

In women, the highest and lowest frequency of disease was found in the people aged 10–19 (n=9, 21.42%) and 1–9 (n=1, 2.38%) yr, respectively. In men, both age groups of 20–29 (n=7, 18.91%) and 50–59 (n=7, 18.91%) yr equally had the highest frequency and the lowest frequency was in the age group of 80–99 yr (n=1, 2.70%).

**Fig. 1:**
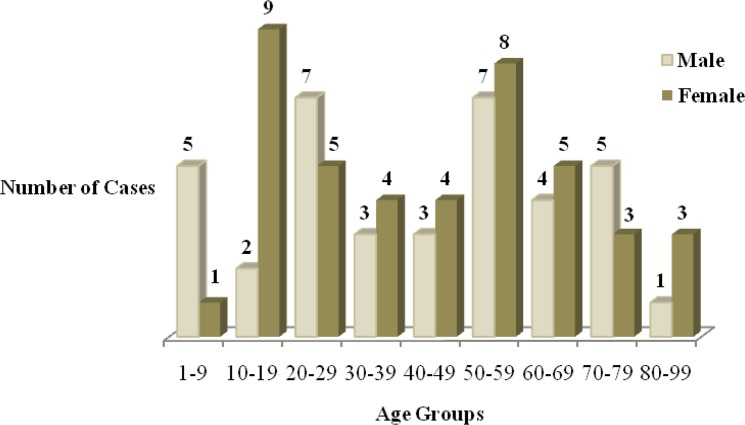
Number of Hydatidosis diagnosed cases by gender and age groups

Totally, the youngest patients were 1–9 yr (n=6, 8%) and the four oldest cases were over 80 yr (n=4, 5%). Moreover, the highest frequency of hydatidosis was between 50–59 yr of age (n=15, 19%) and the lowest frequency was for age more than 80 (n=4, 5%) and then 1–9 yr old (n=6, 8%) (Fig. 1).

A major proportion of presenting symptoms/signs was gastrointestinally related (45 patients). The other cases had pulmonary (n=16), neurologic (n=3) and urologic (n=2) chief complaints. Eleven cases had no symptoms and were incidental.

In our patients, the diagnosis of CE (through clinical and imaging investigations) was established before surgery except for one case in which the patient underwent surgery with primary diagnosis of liver mass and then pulmonary hydatid cyst was confirmed.

The liver was engaged in approximately two-thirds of the cases (n=54). Spleen involvement was in the second place (n=24). Moreover, a small proportion of cases had simultaneous infection of the liver and lungs or extra hepato-pulmonary organs involvement (n=9 or 1.1% for both conditions). Only 4 patients (6%) were diagnosed as secondary hydatid cyst in which the main primary affected organs were liver and lungs. Furthermore, in one case, the primary infection of brain led to involvement of lungs, liver, and pancreas.

The largest and the lowest diameter were 15 and 2 centimeter, respectively and the number of cysts varied from 1 to 4. Most of them had a single hydatid cyst, whereas only 5% had 4 cysts.

Albendazole (15 mg/kg/day divided bd for 6 wk) was prescribed for 47 individuals and it was the only therapeutic approach in 7 cases. Moreover, percutaneous puncture aspiration injection reinjection (PAIR) technique was applied for 6 cases. Sixty-six patients underwent surgery. No data was available on eosinophilic count or serological tests.

## Discussion

Nowadays, CE has become a re-emerging disease and a public health problem of increasing concern around the world (13). Therefore, periodic epidemiological investigation in medical institutions seems to be reasonable. This study focused retrospectively on epidemiological and clinical characteristics of 79 CE patients. The annual frequency of these cases showed an ascending trend in the middle half of the study. This finding can be due to the implementation of the family physician plan in rural and urban areas from 2004.

In this study, the highest and lowest prevalence of CE were between 51–60 yr and more than 80 yr of age, respectively. These findings are consistent with results of a systematic review (14). In hospital studies, young adults and middle-aged individuals were made up the largest proportion of surgical cases. In older individuals of a population, due to long-term but asymptomatic infection, the main features of human cystic hydatidosis may be observed in community studies more than hospital ones (14). Seeking medical attention was less in older patients due to economic problems or disability (15).

In this study, more than half of the subjects were women (M/F ratio=0.88, 47% vs. 53%). A similar finding was reported in a study on surgical cases of CE (16). In this regard, some reasons such as genetic differences, cleaning and eating raw vegetables, geophagy in pregnancy, close contact with domestic animals and cleaning their living space have been discussed (14, 16).

Based on our data, most of the cases were urban residents. Among the hospital-based surveys of CE in Iran, a similar condition has been documented in districts such as Tehran, Kermanshah, and Kashan (17–19). On the other hand, the most common referral cases in provinces of Hamadan and West and East Azerbaijan were rural residents (16, 20, 21). Close contact with soil, cattle, and dogs cause higher prevalence of disease in rural areas (21). In our opinion, higher frequency of CE in urban areas is related to lifestyle of the population in which unhealthy behaviors leading to hydatidosis are being common.

Majority of hydatid cysts were caught from the liver and lungs. This finding is consistent with global epidemiological data in which liver was the most frequently affected organ, in about three-quarters of the cases (22). Other organ operations in our study included spleen, pancreas, mesenteric area, pelvic area, kidney, spine, and CNS. In accordance with the mentioned organs, the most common presenting sign and symptoms were gastrointestinal, urologic and neurologic signs. Except for skin, hair, and nails, CE can be found in all organs of the body (23). For example, there are some reports of primary hydatidosis in orbital intraosseous, cardiac and uterine (24–26).

We had no data about using immunodiagnostic techniques in this study. Although there are some reports on developing high accuracy serological test in laboratory settings such as AgB-based tests. None of the available immunodiagnostic methods are accepted by clinical physicians due to a relatively low sensitivity and specificity (27, 28).

Antiparasitic agents, including Albendazole or Mebendazole, were used for nearly three-fifths of the patients. Appropriate medical therapy before and after surgery may lead to fewer relapses (29, 30). Moreover, in patients with several cysts, combination therapy with Albendazole and Praziquantel can be an alternative to surgery (29, 31).

In our setting, surgical resection and open complete cyst removal with installation of a scolicidal agent was the preferred technique. The conservative procedures consisted of drainage, marsupialization and puncture aspiration injection reinjection technique. No laparoscopic surgery was registered. In a review of laparoscopic treatment of liver cystic echinococcosis, due to increasing the number of surgeons, the application of this procedure will be more adopted (22). Safety of the patient and cystic integrity for prevention of the spillage of cystic contents is of importance (32). Recently, by assembling disposable, cheap, and available anesthesia equipment with common laparoscopic instruments, a simple and safe laparoscopic hydatid evaluation system has been introduced in Iran (33).

Our study had some limitations. First, it was a retrospective review of hospital records with a risk of information bias based on the quality of recording and extraction of information. Second, no follow up information was available and in about one-third of the patients, the diameter and number of cysts were not recorded. Designing and developing a registry system for recording all CE cases diagnosed in Iran with a unique code for every patient, encompassed epidemiological and medical data, will improve the condition of recording in hospital charts. In addition, it will be used as a national reference dataset enabling a dynamic surveillance system, including follow-up data as well as environmental and parasitological data. In this regard, developing a new data management system for the French National Registry of human alveolar echinococcosis (AE) cases can be considered (34). This system is responsible for recording all AE cases diagnosed in France. Administrative, epidemiological and medical information of the patients are recorded. The current data set will evolve towards a dynamic surveillance system, including follow-up data (e.g. imaging, serology) and will be connected to environmental and parasitological data to better understand the pathogen transmission pathway.

## Conclusion

CE is always relatively prevalence in Iranian communities and prevention and control programs run continuously. Development of new diagnostic methods and therapeutic procedures is worthy. Moreover, it is needed to design and develop a registry and surveillance system by a multidisciplinary team.
